# Acceptability of oral liquid pharmaceutical products in older adults: palatability and swallowability issues

**DOI:** 10.1186/s12877-019-1337-2

**Published:** 2019-12-07

**Authors:** Emilie Belissa, Thibault Vallet, Sandra Laribe-Caget, Alain Chevallier, François-Xavier Chedhomme, Fattima Abdallah, Nathalie Bachalat, Sid-Ahmed Belbachir, Imad Boulaich, Vanessa Bloch, Anne Delahaye, Mathieu Depoisson, Amélie Dufaÿ Wojcicki, Stéphane Gibaud, Anne-Sophie Grancher, Caroline Guinot, Celia Lachuer, Laurent Lechowski, Patrick Leglise, Abdel Mahiou, Sylvie Meaume, Corinne Michel, Hugues Michelon, Yann Orven, Ines Perquy, Matthieu Piccoli, Maïté Rabus, Annie-Claude Ribemont, Jean-Paul Rwabihama, Jean-Hugues Trouvin, Fabrice Ruiz, Vincent Boudy

**Affiliations:** 1 Département Recherche et Développement Pharmaceutique, Agence Générale des Equipements et Produits de Santé (AGEPS), Assistance Publique-Hôpitaux de Paris (AP-HP), 7 rue du Fer à Moulin, 75005 Paris, France; 2 ClinSearch, 110 avenue Pierre Brossolette, 92240 Malakoff, France; 3 Hôpital Rothschild, Groupe Hospitalier Universitaire Est Parisien, AP-HP, 5 rue Santerre, 75012 Paris, France; 4 Hôpital Broca, Groupe Hospitalier Universitaire Paris Centre, AP-HP, 54-56 rue Pascal, 75013 Paris, France; 5 Hôpital Joffre Dupuytren, Groupe Hospitalier Universitaire Henri Mondor, AP-HP, 1 rue Eugène Delacroix, 91210 Draveil, France; 6 Hôpital René Muret, Groupe Hospitalier Universitaire Paris Seine-Saint-Denis, AP-HP, avenue du Dr Schaeffner, 93270 Sevran, France; 7 Hôpital Fernand Widal, Groupe Hospitalier Universitaire Saint-Louis – Lariboisière – Fernand-Widal, AP-HP, 200 rue du Faubourg Saint-Denis, 75010 Paris, France; 8 Hôpital Sainte Périne, Groupe Hospitalier Universitaire Paris Ile-de-France Ouest, AP-HP, 11 rue Chardon Lagache, 75016 Paris, France; 9 Hôpital Vaugirard, Groupe Hospitalier Universitaire Paris Ouest, AP-HP, 10 rue Vaugelas, 75015 Paris, France; 10 Centre Hospitalier de l’Ouest Vosgien, 1280 avenue division Leclerc, 88300 Neufchâteau, France

**Keywords:** Medicine acceptability, Older population, Palatability, Swallowability, Oral liquids

## Abstract

**Background:**

In institutional care, oral liquid pharmaceutical products are widely prescribed for older patients, especially for those with swallowing disorders. As medicines acceptability is a key factor for compliance in the older population, this study investigated the acceptability of oral liquid pharmaceutical products in this targeted population.

**Methods:**

An observational, multicenter, prospective study was conducted in eight geriatric hospitals and eight nursing homes in France. Observers reported several behaviours/events describing the many aspects of acceptability for various pharmaceutical products’ uses in patients aged 65 and older. Acceptability scores of oral liquid pharmaceutical products were obtained using an acceptability reference framework (CAST - ClinSearch Acceptability Score Test®): a 3D-map summarizing the different users’ behaviors, with two clusters defining the positively and negatively accepted profiles materialized by the green and red zones, respectively.

**Results:**

Among 1288 patients included in the core study and supporting the acceptability reference framework, 340 assessments were related to the administration of an oral liquid pharmaceutical product. The mean age of these patients was 87 (Range [66-104y]; SD = 6.7), 68% were women and 16% had swallowing disorders. Globally, the oral liquid pharmaceutical products were classified as “positively accepted,” the barycenter of the 340 assessments, along with the entire confidence ellipses surrounding it, were positioned on the green zone of the map. Sub-populations presenting a different acceptability profile have also been identified. For patients with swallowing disorders, the oral liquid pharmaceutical products were classified as “negatively accepted,” the barycenter of the 53 assessments along with 87% of its confidence ellipses were associated with this profile. A gender difference was observed for unflavored oral liquids. In women, they were classified “negatively accepted,” the barycenter of the 68 assessments with 75% of its confidence ellipses were located in the red zone, while they were classified “positively accepted” in men.

**Conclusion:**

This study showed that oral liquid pharmaceutical products are a suboptimal alternative to solid oral dosage forms in patients with swallowing disorders. To ensure an optimal acceptability, prescribers should also consider the presence of a taste-masker in these oral liquids. As highlighted herein, palatability remains crucial in older populations, especially for women.

## Background

Nearly 10% of older patients are hospitalized owing to non-compliance to their daily intake of prescribed medications [1]. Medicine acceptability is likely to have a significant impact on patient adherence in these older populations and consequently on the efficiency and safety of treatments as well as overall quality of life. Patient acceptability in the older population has been recently defined by the European Medicines Agency (EMA) as “the ability and willingness of the patient to self-administer and also any of their lay or professional caregivers, to administer the medicinal product as intended” [2]. It is a multidimensional concept, depending on characteristics of the drug product – such as route of administration, appearance, swallowability, and characteristics of the patients themselves – such as age and pathological state, in both pediatric and older populations [2, 3]. To ensure medicine acceptability for each of these populations, appropriate pharmaceutical products must be developed [4, 5].

Altered swallowing function is a common disability concerning 2 to 16% of the community-dwelling older population, affecting up to 60% of patients in some institutions [6, 7]. This age-related swallowing impairment is a major issue compromising the use of the most prescribed formulation in older patients, the solid oral dosage form (SODF). Consequently, SODF are frequently subject to alterations, mainly crushing, at the time of administration [8]. Although dysphagia may be diagnosed using the drink test, measuring the time needed to swallow 80 mL of water [7], recourse to oral liquid pharmaceutical products is a widely used alternative to SODF in an attempt to ease medicine administration for these patients with swallowing disorders [9]. Despite the many well-known drawbacks for the older population (e.g., risk of dosage errors or an incomplete administration, risk of excipient overload [2]), switching from SODF to oral liquid formulations nonetheless remains a common practice in institutional care.

Oral liquid pharmaceutical products encompass ready-to-use oral liquids (i.e., oral solutions or suspensions) as well as the reconstituted oral liquids (i.e., powders or effervescent tablets which must be dissolved or dispersed in a liquid prior to administration). Many active pharmaceutical ingredients (API) have a bitter taste [10], for which excipients are frequently used as masking agents. Although palatability is known to be an important acceptability driver in pediatrics [3], it is usually not considered to be a major issue for older patients in hospitals and nursing homes, due to the increasing prevalence of dysgeusia with age, and polymedication in this population [11–16].

This study investigated the impact of both users and products characteristics on acceptability of oral liquid pharmaceutical products in such an older population.

## Methods

### Objective, study design and setting

The objective of this multicenter, prospective, cross-sectional, and strictly observational study, was to identify those pharmaceutical products less accepted in the older population. The study was conducted between October 2016 and August 2018 in 8 French hospitals and 8 nursing homes.

### Participants

Inclusion criteria required that patients were 65-years-old and older, hospitalized or residing in a nursing home, receiving any medicine. Patients receiving intravenous medication where the intravenous device is already in situ were excluded, as the insertion of such a device was considered as part of the acceptability. All patients answering these criteria, and who have assented verbally to participating in the study, were included without any randomization. The observer reports were recorded anonymously. Being a non-interventional study, patients were maintained under their current treatment, no modifications or additions to their prescriptions were made during the observational period.

### Data collection

CAST - ClinSearch Acceptability Score Test®, is a tool integrating many aspects of acceptability to discriminate between positively and negatively accepted medicines in vulnerable populations. CAST was initially developed for the pediatric population [17, 18], and has since been transposed for the older population [19].

This tool includes observer reports corresponding to several observations of behaviors/events performed during the medicine use: patient’s reaction during the administration (positive, neutral or negative reaction), result of the administration (the dose is fully, partly or not taken at all), time needed to prepare (from opening any packaging to having a required dose ready to use, including all handling and modifications), and time to administer the required dose of medication (from a required dose of medication ready to use to the end of the intake). The preparation and administration time was classified as 20 s or shorter, medium time, or longer than one minute. In addition, observers were required to report recourse to any methods used to ease/achieve administration. This might include dividing the intake of a dose which cannot be taken as a whole; altering the intended use (modify the dosage form such as tablet crushed or capsule opened; use another route/mode of administration; use a device not provided); use of food/drink to mask a taste or ease swallowing; use of restraint (the patient had to force himself or was opposed taking the medication). These observational variables were collected prospectively by observers in order to score acceptability.

Each evaluation of one medication taken by one patient corresponding to a particular combination of observed measures was related to information on the product, the patient and the context of use [19], as described briefly hereafter. Demographic parameters (e.g. sex) and comorbidities (e.g. swallowing disorders) were recorded from the patient’s medical record, while information on the pharmaceutical products (e.g. pharmaceutical form, presence of flavoring agent) were extracted from the summary of product characteristics (SmPC). These explanatory variables were collected in order to investigate their impact on acceptability.

### Statistical analyses

To describe the many aspects of acceptability, previously described observations were included in a multivariate analysis without weighting. Using mapping and clustering processes, an intelligible acceptability reference framework was designed: a three-dimensional acceptability map was created that juxtaposed two clusters of evaluations, each defining a contrasting acceptability profile. All of the evaluations were positioned on the map depending on their similarity: the most similar evaluations converged. Positively connoted observations were over-represented in the first cluster of evaluations defining the “Positively accepted” profile (green area on the map), while negatively connoted evaluations were over-represented in the second cluster defining the “Negatively accepted” profile (red area on the map).

The reference framework was used to investigate the impact of characteristics of both users (gender and presence of swallowing alteration) and drug products (presence of flavoring agents) on acceptability of the oral liquid pharmaceutical products.

To obtain an acceptability score the barycenter of the evaluations of interest (e.g. those related to the flavored formulations) was positioned on the map. A barycenter, along with the entire 90% confidence ellipses surrounding it, belonging to the “positively accepted” profile, could be considered as accepted. Due to differences among patients, we consider a minimum of 30 evaluations to get a robust acceptability score. In cases with fewer than this threshold, we cannot draw any conclusion and only note any tendency that might be observed.

Pearson’s Chi-squared test was used to assess the significance of the differences observed between the subpopulations of patients related to the different acceptability scores, in terms of gender and swallowing disorders [20].

The R packages “FactoMineR” [21] and “MissMDA” [22] were used to perform multivariate analysis and to handle missing data, respectively (R version 3.4.4).

## Results

### Patients and medicines

Among the 1288 evaluations included in the multivariate analysis that gave rise to the final acceptability reference framework, there were 340 evaluations of oral liquid pharmaceutical products. The mean age of these patients was 87 (6.7), the minimum age was 66, the maximum was 104, and 68% were women. Thirty five percent of patients had taken ready-to-use oral liquids and the remaining 65% had taken reconstituted oral liquids. Table 1 presents the demographic characteristics of these patients and the features of the 59 distinct pharmaceutical products are summarized in Table 2.

**Table 1:** Demographic Characteristics of the Patients

Characteristics of patients (*n* = 340)		n	(%)
Sex	Women	230	(68)
	Men	107	(32)
	md^a^: 3		
Age (years)	[65, 75]	13	(4)
	[75, 85]	111	(33)
	[85, 95]	172	(50)
	[95, 104]	43	(13)
	md: 1		
Place	Hospital	304	(89)
	Nursing home	36	(11)
Disabilities	Swallowing disorder	53	(16)
	md: 4		
	Muscular or rheumatologic disorders of the upper limbs	81	(24)
	md: 6		
	Cognitive impairment	210	(62)
	md: 4		
Number of prescribed medicines per day	1–4	31	(9)
	5–9	147	(43)
	≥10	161	(48)
	md: 1		

^a^*md* missing data

**Table 2:** Characteristics of the Medicines

Characteristics of medicines (*n* = 59)		n	(%)
Formulations	Oral solution	17	(29)
	Powder for oral solution	11	(19)
	Oral suspension	6	(10)
	Powder for oral suspension	4	(7)
	Drops for oral solution	4	(7)
	Divisible effervescent tablet	3	(5)
	Dispersible tablet	3	(5)
	Other (2% < *n* < 5%): Syrup; Effervescent tablet.		
	Other (*n* ≤ 2%): Powder for oral and rectal solution; Oral gel; Injection and oral solution; Prolonged release granules; Divisible tablet for oral suspension; Dispersible or chewable tablet; Dispersible coated tablet		
Flavoring agents	Presence	40	(70)
	Absence	17	(30)
	md^a^: 2		
Therapeutic subgroups (ATC^b^ - 2nd level)	Analgesics	10	(17)
	Drugs for constipation	8	(14)
	Psycholeptics	7	(12)
	Antiepileptics	7	(12)
	Psychoanaleptics	4	(7)
	Mineral supplements	4	(7)
	Antibacterials for systemic use	4	(7)
	Other (2% < n < 5%): Drugs used in diabetes; Drugs for acid related disorders; Antithrombotic agents		
	Other (n ≤ 2%): Thyroid therapy; Beta blocking agents; Anti-parkinson drugs; All other non-therapeutic products; Antimycotics for systemic use; Antimycobacterials; Antihemorrhagics; Antianemic preparations		

^a^*md* missing data

^b^ATC: The Anatomical Therapeutic Chemical (ATC) Classification System is a drug classification system controlled by the World Health Organization Collaborating Centre for Drug Statistics Methodology (WHOCC)

### Acceptability of oral liquid pharmaceutical products

The barycenter of the 340 evaluations of oral liquid pharmaceutical products, along with the entire 90% confidence ellipses surrounding it, fell within the “positively accepted” profile cluster. Thus, the oral liquid pharmaceutical products, considered as a whole, were classified as accepted in the older population.

### Effect of swallowing alteration in patients

**Fig. 1:**
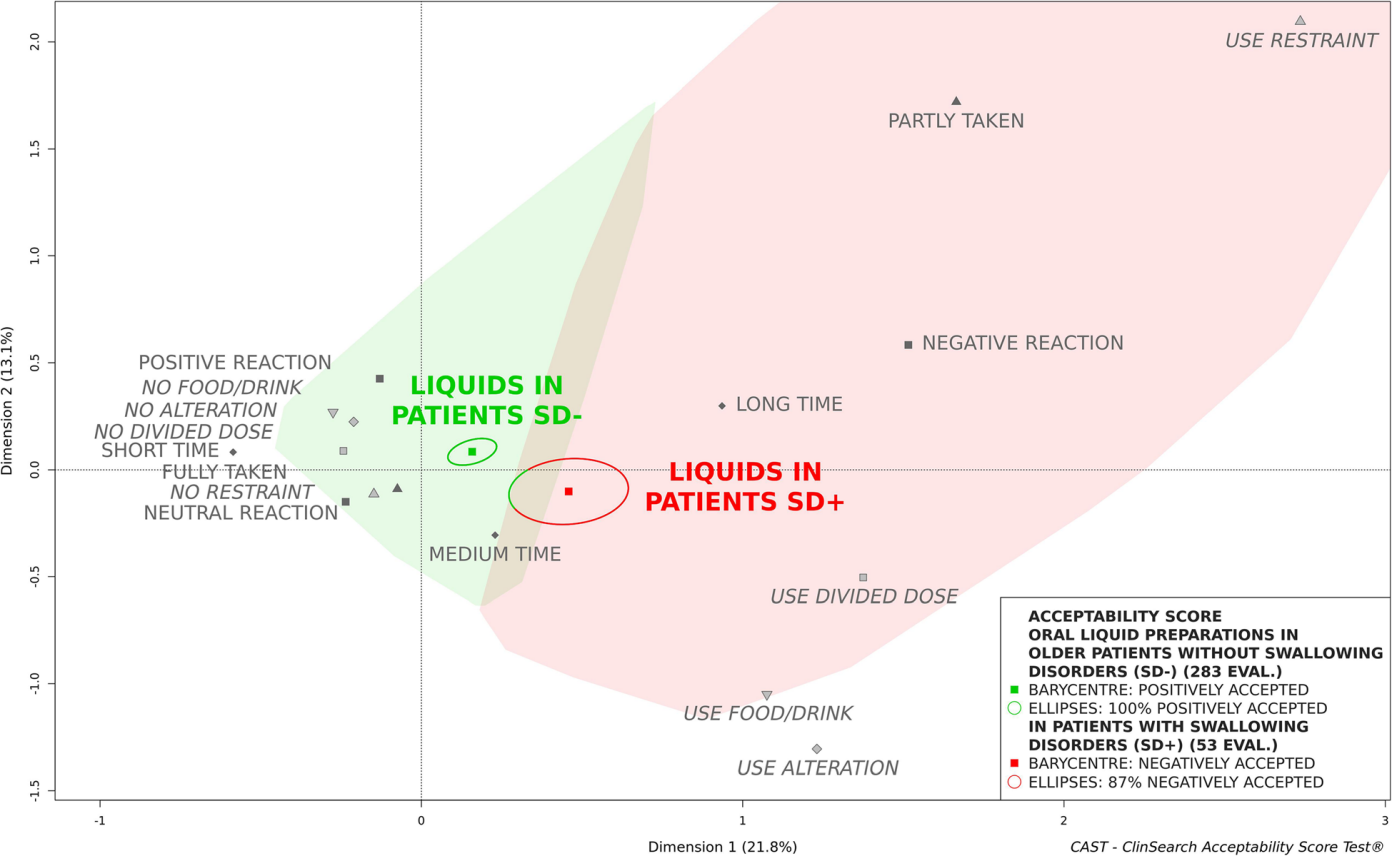
Acceptability profiles of oral liquid pharmaceutical products in the older patients with (SD+) and without (SD-) swallowing disorders

Among all the patients who had taken oral liquid pharmaceutical products, 16% had a swallowing disorder. (Figure 1) presents the acceptability scores of oral liquid pharmaceutical products in patients with or without swallowing disorders. This figure highlights the negative impact of dysphagia on the acceptability of such liquid formulations, which could be classified as accepted in patients without a swallowing disorder but not in patients with swallowing disorders.

There were no significant differences between these groups of patients in term of sex (*p* = 0.999) flavored formulation (*p* = 0.877).

### Effect of flavoring agents and gender of patients

**Fig. 2:**
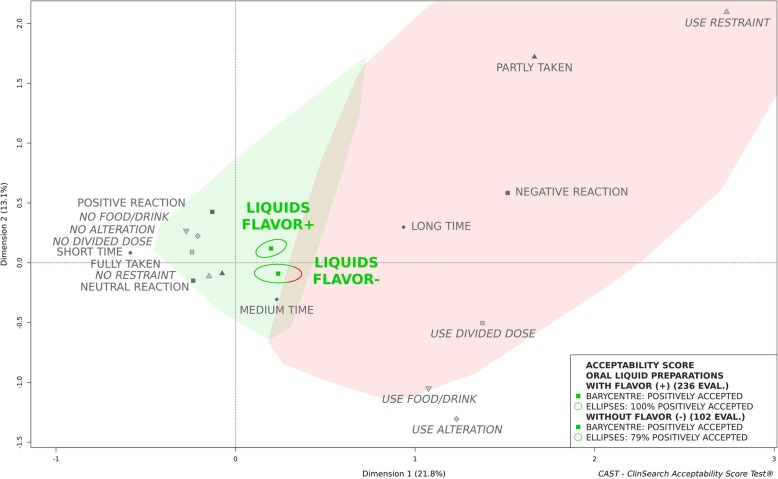
Acceptability profiles of oral liquid pharmaceutical products with (FLAVOR+) and without (FLAVOR-) flavoring agents

A third of the patients (*n* = 102) had taken oral liquids pharmaceutical products that had no flavoring agents added. (Figure 2) presents the acceptability scores of the products formulated with or without flavoring agents. Figure 2 highlights the positive impact of flavoring on the acceptability of these formulations. Those formulated with a flavoring agent could be classified as accepted, while those formulated without any flavoring agent could not, due to a significant part of their confidence ellipses falling within the second cluster.

There were no significant differences between these groups of patients in term of sex (*p* = 0.814) or swallowing disorders (*p* = 0.877).

**Fig. 3:**
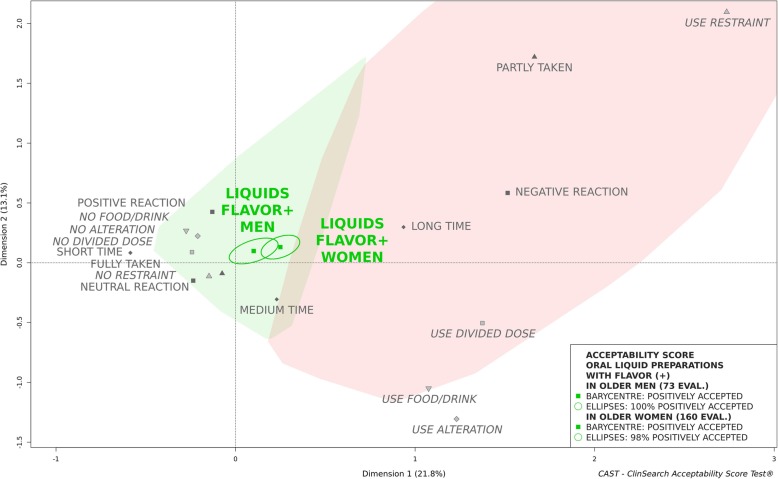
Acceptability profiles of oral liquid pharmaceutical products with flavor (FLAVOR+) in men and women

**Fig. 4:**
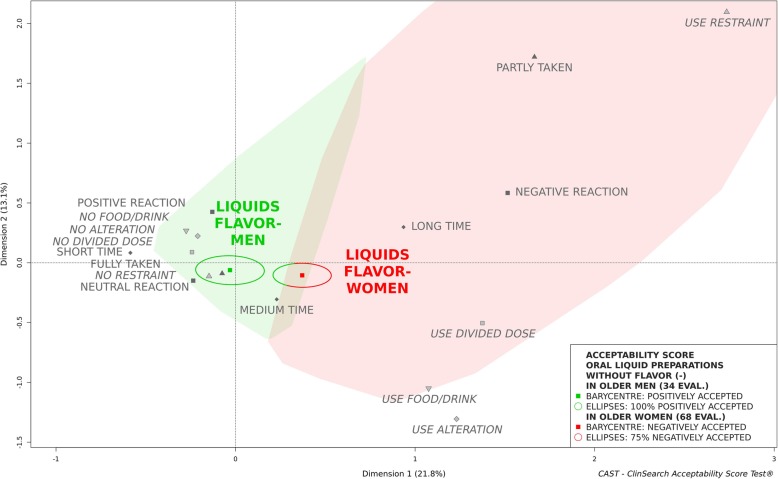
Acceptability profiles of oral liquid pharmaceutical products without flavor (FLAVOR-) in men and women

Figures 3 and 4 investigate palatability issues depending on patients’ gender. The oral liquids formulated with flavor seemed to be accepted regardless of patients’ gender, as the barycentre, along with the entire confidence ellipses, with the exception of a very limited part of the ellipse in the third dimension for women, belonged to the “positively accepted” profile (Fig. 3). While those formulated without flavor could be classified as accepted in men but not in women (Fig. 4).

There were no significant differences between men and women in terms of swallowing disorders for the oral liquids formulated with flavor (*p* = 0.732) and without flavor (*p* = 0.661).

## Discussion

The population studied, receiving pharmaceutical products under the specific dosage form of oral liquids, is representative of the older population classically encountered in hospitals or nursing homes. Most patients were women over 80 years old, a consequence of the longer life expectancy of women [1, 23]. In this study’s population, polymedication of greater than 5 medication per day was observed among 91% of the patients, which is higher than that previously reported for older populations, e.g., 50% in the studies of Legrain [1] and Solemdal [13]. However, this could in part be due to the fact that in this study the prescription of “in case of” medications, which is very frequent in the hospital settings, have also been counted for each patient. To avoid or limit any inclusion or selection bias, all patients meeting the inclusion criteria were proposed to participate. Twenty six percent of the patients included in the core study had taken oral liquid pharmaceutical products. Although these products, considered as a whole, were classified as accepted in the older population using the reference framework, this study has revealed acceptability differences driven by both users and products characteristics.

Oral liquid pharmaceutical products were classified as accepted in older patients without any swallowing disorders, while this appeared not to be the case for those with swallowing alterations. These results confirm that recourse to these formulations in the older population with swallowing alterations remains a suboptimal alternative to SODF. As age-related swallowing impairments are nonetheless a common disability affecting oral medicines administration, further investigations are carried out to provide healthcare professionals with relevant knowledge on which formulations (e.g. orodispersible forms) and medicine features (e.g. maximum size of a tablet) ensure an optimal acceptability in these patients.

Oral liquids formulated without any addition of a flavoring agent appeared to be less well accepted than those that were flavored. Highlighting the positive impact of flavoring agents on the acceptability of oral liquid formulations, these results demonstrate the critical aspect of pharmaceutical products’ palatability in the older population. In pediatrics, the importance of palatability is well-known [3], have shown that flavors may have a favorable effect on medicines acceptability [24–28]. In the older population, palatability has often been overlooked and commonly overshadowed by swallowability issues. Indeed, initial drafts of the EMA reflection paper on the pharmaceutical development of medicines for use in the older population did not mention palatability as a product characteristic influencing medicines acceptability in the older population [2].

Exploring gender acceptability differences, a higher sensitivity among women to the unpalatable oral liquids has been observed. Indeed, contrary to those formulated with flavor which were accepted regardless of patients’ gender, oral liquids formulated without any additional flavoring agent were classified as accepted in older men, but not in women. This may be supported by the fact that women usually maintain a higher taste perception than their male counterparts over the course of ageing [11–13, 16]. Therefore, palatability was found to be a key factor of influence upon acceptability, especially for older women.

Further explorations of factors impacting palatability are conducted because other characteristics of patients such as health status (e.g. dementia [29]) could affect smell and taste perception and consequently, medicine acceptability.

Among the 59 distinct pharmaceutical products assessed in this study, 30% were formulated without any flavoring agents. Overcoming API taste issues through the judicious choice of excipients seems appropriate to ensure liquid preparations acceptability in the older population. However, the potential benefits of excipients should be tempered with regards to safety concerns (e.g. sugars may negatively impact oral health and increase blood glucose levels) as well as particular conditions and any other prescriptions associated with ageing (e.g. multiple long-term therapies resulting in polyols overload may have a laxative effect). Furthermore, the use of certain sweeteners and flavoring agents could be challenging due to issues of stability and/or compatibility with the API in question or other excipients. A better understanding of patients’ needs and challenges in elderly formulation development is thus required for the prescription, and development, of dosage forms that are appropriate for each individual.

## Conclusion

This study has demonstrated in an objective manner that both swallowability and palatability issues remain crucial for the acceptability of oral liquid pharmaceutical products in older people hospitalized or receiving institutional care. Indeed, these products are a suboptimal alternative to solid oral dosage forms in patients with swallowing disorders, while their palatability remains an essential acceptability driver in older adults, especially in women. These findings underline the need to promote patient-centered care based on a better understanding of the older patient’s needs.

## Data Availability

Data underlying the study cannot be made publicly available due to legal and ethical considerations. European Union (GDPR) and French (Law n°78–17 of 6 January 1978) laws restrict the public sharing of personally identifiable data. Requests for data will be processed according to the French MR-003 Code of conduct by the data controller, ClinSearch, which allows for the use of data for the purpose of reproducing study results. Requests to access the data for this purpose may be sent to the data protection officer of ClinSearch: dataprivacy@clinsearch.net, and researchers outside the European Union will need to sign a transfer agreement.

## Abbreviations

API: Active pharmaceutical ingredients
CAST: CAST - ClinSearch Acceptability Score Test®
EMA: European medicines agency
SmPC: Summary of product characteristics
SODF: Solid oral dosage form
